# Screening for Fabry Disease in patients with unexplained left ventricular hypertrophy

**DOI:** 10.1371/journal.pone.0239675

**Published:** 2020-09-28

**Authors:** Chandu Sadasivan, Josie T. Y. Chow, Bun Sheng, David K. H. Chan, Yiting Fan, Paul C. L. Choi, Jeffrey K. T. Wong, Mabel M. B. Tong, Tsz-Ngai Chan, Erik Fung, Kevin K. H. Kam, Joseph Y. S. Chan, Wai-Kin Chi, D. Ian Paterson, Manohara Senaratne, Neil Brass, Gavin Y. Oudit, Alex P. W. Lee

**Affiliations:** 1 Department of Medicine, University of Alberta, Edmonton, Canada; 2 Mazankowski Alberta Heart Institute, University of Alberta, Edmonton, Canada; 3 Division of Cardiology, Department of Medicine and Therapeutics, The Chinese University of Hong Kong, Hong Kong SAR, China; 4 Princess Margaret Hospital, Hong Kong SAR, China; 5 Clinical Genetic Service, Department of Health, Hong Kong SAR, China; 6 Laboratory of Cardiac Imaging and 3D Printing, Li Ka Shing Institute of Health Science, Hong Kong SAR, China; 7 Department of Anatomical and Cellular Pathology, The Chinese University of Hong Kong, Hong Kong SAR, China; 8 Department of Imaging and Interventional Radiology, Hong Kong SAR, China; 9 Department of Radiology, Alice Ho Miu Ling Nethersole Hospital, Hong Kong SAR, China; 10 Division of Cardiology, Grey Nuns Community Hospital, Edmonton, Alberta, Canada; 11 Division of Cardiology, Royal Alexandra Hospital, Edmonton, Alberta, Canada; King's College London, UNITED KINGDOM

## Abstract

Fabry Disease (FD) is a systemic disorder that can result in cardiovascular, renal, and neurovascular disease leading to reduced life expectancy. FD should be considered in the differential of all patients with unexplained left ventricular hypertrophy (LVH). We therefore performed a prospective screening study in Edmonton and Hong Kong using Dried Blood Spot (DBS) testing on patients with undiagnosed LVH. Participants found to have unexplained LVH on echocardiography were invited to participate and subsequently subjected to DBS testing. DBS testing was used to measure α-galactosidase (α-GAL) enzyme activity and for mutation analysis of the α-galactosidase (*GLA*) gene, both of which are required to make a diagnosis of FD. DBS testing was performed as a screening tool on patients (*n* = 266) in Edmonton and Hong Kong, allowing for detection of five patients with FD (2% prevalence of FD) and one patient with hydroxychloroquine-induced phenocopy. Left ventricular mass index (LVMI) by *GLA* genotype showed a higher LVMI in patients with IVS4 + 919G > A mutations compared to those without the mutation. Two patients were initiated on ERT and hydroxychloroquine was discontinued in the patient with a phenocopy of FD. Overall, we detected FD in 2% of our screening cohort using DBS testing as an effective and easy to administer screening tool in patients with unexplained LVH. Utilizing DBS testing to screen for FD in patients with otherwise undiagnosed LVH is clinically important due to the availability of effective therapies and the value of cascade screening in extended families.

## Introduction

Fabry Disease (FD) is an X-linked lysosomal storage disease, where a deficiency in the enzyme α-galactosidase (α-GAL) results in the accumulation of its substrate, globotriaosylceramide (Gb3), which is a glycosphingolipid [1,2]. FD is a systemic disorder that results in heart disease, renal failure, neurovascular disease, and various other systemic manifestations as a result of Gb3 accumulation [3,4]. Cardiovascular disease is the primary cause of death in patients with FD and cardiac manifestations of the disease include left ventricular hypertrophy, diastolic dysfunction, valvular abnormalities, and conduction abnormalities [4–7]. Patients with FD have a reduced life expectancy, characterized by a reduction of 20 years in male carriers and 15 years in female carriers compared to the compared to the general population [8,9].

While the clinical diagnosis of FD is challenging, the biochemical basis for FD diagnosis can be made easily based on decreased leukocyte α-GAL A activity in males and for females that have a normal enzyme level, mutation analysis of the alpha-galactosidase gene (*GLA*) using the dried blood spot (DBS) testing [4,5]. There are various reasons to establish an early diagnosis of FD. First, there have been advances in the use of enzyme replacement therapy (ERT) as a treatment for FD and it is available in Hong Kong and Canada for patients who meet the criteria [10–12]. Second, FD associated myocardial fibrosis, a substrate for arrhythmias, is considered to be irreversible and unresponsive to ERT, clearly supporting the need for early diagnosis in these patients [13,14]. Third, ERT and chaperone therapy are efficacious in reducing left ventricular mass and hypertrophy [15–17]. Fourth, applying a precision medicine approach to diagnosis and therapy will improve clinical outcomes for patients with FD, while also providing risk stratification for family members if a pathogenic variant is identified [18]. The current study was designed to assess whether DBS testing may be used to diagnose FD, allowing for earlier detection of the disease in patients with left ventricular hypertrophy (LVH) and to allow for treatment to be started promptly.

## Methods

This study was approved at the University of Alberta Health Research Ethics board (Pro00058901) and the joint Chinese University of Hong Kong-New Territories East Cluster Clinical Research Ethics Committee (CRE-2017.112). Written consents were obtained from all participants.

### Participants

**Fig 1:**
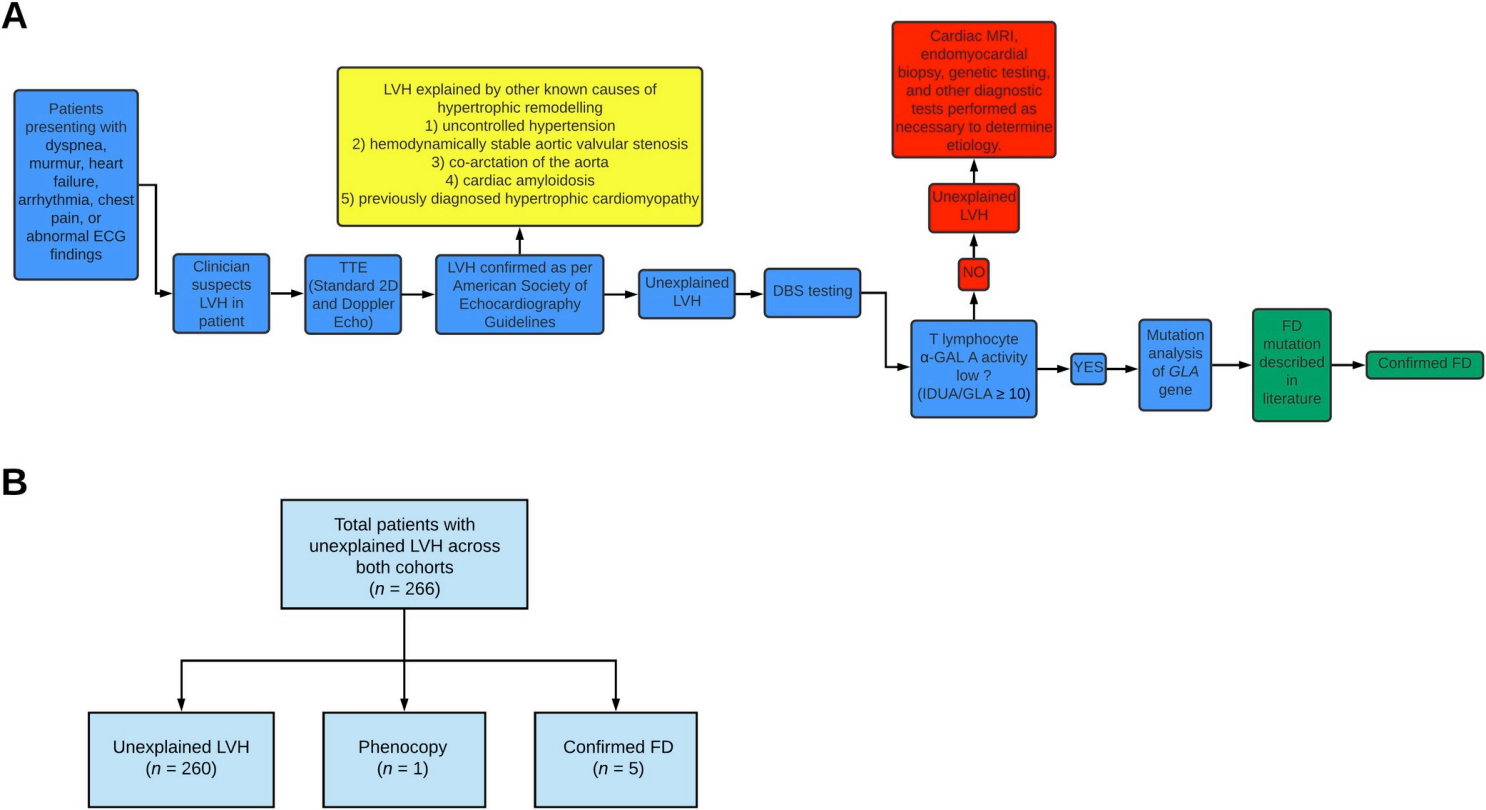
(A) Schematic showing overall design of study and flow of patients across both sites. (B) Flow diagram depicting breakdown of patients across both Edmonton and Hong Kong cohorts. LVH indicates left ventricular hypertrophy; TTE, transthoracic echocardiography; DBS, dried blood spot; a-GAL A, α-galactosidase A enzyme; IDUA, alpha-L-iduronidase; GLA, α-galactosidase gene; FD, Fabry Disease; HCQ, hydroxychloroquine.

Echocardiography was performed on patients presenting with dyspnea, murmurs, heart failure, arrhythmia, chest pain, or abnormal ECG findings. Patients who were prospectively identified to have left ventricular hypertrophy (LVH) on echocardiography were then recruited to participate in the study at both Hong Kong and Edmonton sites (Fig 1A). Our study was conducted at the Prince of Wales Hospital (Hong Kong) and at the University of Alberta Hospital (Edmonton, Alberta, Canada); after appropriate ethics approval was obtained from the appropriate institutional review board. Participants were also informed about FD, how the diagnosis of the disease is made, and the impact for family and the treatment options available. Inclusion criteria for the study included the following: 1) ability to provide informed consent, 2) LVH based on echocardiography. Participants were excluded from the study if other known causes of hypertrophic remodeling were present in their clinical history, such as the following: 1) uncontrolled hypertension, 2) hemodynamically stable aortic valvular stenosis, 3) coarctation of the aorta, 4) cardiac amyloidosis, and 5) previously diagnosed hypertrophic cardiomyopathy. Patients that were pregnant or suspected to be pregnant were also excluded. Cardiac magnetic resonance imaging was performed when appropriate.

### Transthoracic echocardiography

Transthoracic echocardiography (TTE) was performed prospectively on patients with suspected LVH. Left ventricular cavity size was described using linear internal dimensions and volumes as per American Society of Echocardiography (ASE) recommendations [19]. Measurements were reported for end-diastole and end-systole and left ventricular mass (LVM) was indexed to body surface area (BSA) to allow for comparison among patients. Additionally, assessment for aortic valvular stenosis was completed following ASE/European Association of Cardiovascular Imaging (EACVI) clinical recommendations [20].

### Testing for enzyme activity and mutations in *GLA* gene

FD screening was prospectively performed on adult patients diagnosed to have LVH based on TTE. LVH was defined using threshold values of LV mass (LVM) indexed to body surface area (BSA) >115 g/m^2^ and 95 g/m^2^ for men and women, respectively, in the absence of increased LV end diastolic dimension (i.e. <58.4 mm for men and <52.2 mm for women) [19]. Standard 2D and Doppler echocardiography were performed. The clinically acceptable standard method of dried blood spot (DBS) testing was utilized to measure T-lymphocyte alpha-galactosidase activity and for DNA extraction and mutation analysis of the *GLA* gene. Enzyme activity was tested in T-lymphocytes as this is the current accepted clinical method internationally [21,22].

In the Hong Kong cohort, for the DBS enzyme assay, alpha-L-iduronidase (IDUA) was used as the control enzyme. Low plasma α-Gal A activity was defined as the IDUA/GLA ratio ≥ 10 [23]. For both males and females, when their α-GAL A level was within the low range as per the above criteria, Sanger Sequencing was used to search for mutations in the *GLA* gene. In the Edmonton cohort, genetic testing looking for mutations in the *GLA* gene was conducted regardless of plasma α-Gal A activity. DNA was extracted from dried blood spots and the sequencing covered all *GLA* exons and up to 20 base pairs upstream and downstream of the intron-exon boundaries at both sites. Due to the high prevalence of the known intronic mutation, IVS4 + 919G > A, in East Asian populations, an additional 3 mL of blood was also collected and kept in an ethylenediaminetetraacetic acid tube for detection of intronic mutations with Sanger Sequencing. Patients with genetically confirmed FD underwent endomyocardial biopsy to obtain tissue confirmation of cardiac involvement of FD and contrast enhanced cardiac MRI (CMR) to assess the extent of myocardial fibrosis as workup for eligibility for ERT in appropriate patients. CMR (1.5 Tesla) was performed using a standard clinical protocol. Myocardial fibrosis was assessed by late gadolinium enhancement (LGE). Delayed images (8 mm slice thickness; both long and short-axis views) was applied to detect LGE 10 minutes after intravenous contrast administration (0.1 mmol/kg body weight, gadoterate meglumine, Dotarem, Guerbet S.A., France). In the Hong Kong cohort, additional clinical information was collected including the presence of classical symptoms of FD including angiokeratoma, acroparesthesias, and hyperhidrosis, and the documentation of ECG changes and arrhythmia.

### Statistical analysis

Statistical analyses were carried out using IBM SPSS Statistics 26.0 for Macintosh OS (SPSS Inc, Chicago, IL). Patient characteristics are reported as mean ± SD or median (range) based on results of the Shapiro-Wilk test of normality. Linear regression analysis was used to compare α-GAL enzyme activity to various left ventricular parameters and Mann-Whitney *U* test was used to compare left ventricular mass index (LVMI) to *GLA* genotype. An independent analysis of the Hong Kong and Edmonton cohort was conducted to verify the robustness of the results. A *P*-value <0.05 was considered statistically significant through all statistical analysis.

## Results

Dried blood spot (DBS) testing was performed as a screening tool in a total of 266 patients from both Edmonton and Hong Kong sites (S1 File). Both Edmonton (*n* = 30) and Hong Kong (*n* = 236) cohorts were comparable to one another in terms of demographic characteristics and left ventricular parameters (Table 1 and Fig 1A). Patients across both screening cohorts had moderate to severe left ventricular hypertrophy as indicated by LVMI. Screening with DBS testing allowed detection of five patients with Fabry Disease (FD) and one hydroxychloroquine (HCQ)-induced phenocopy (Fig 1B). To determine the cause of left ventricular hypertrophy in the remaining patients, cardiac MRI, endomyocardial biopsy, genetic testing, and other diagnostic testing were performed as necessary as an integral part of the clinical diagnostic investigation.

**Table 1:** Patient demographics and echocardiographic parameters in screening cohorts. Data is expressed as mean ± SD or median (range) if data is not normally distributed (Shapiro-Wilk, *P*<0.05).

Patient Characteristics	Hong Kong (*n* = 236)	Edmonton (*n* = 30)	Total (*n* = 266)
Age (years)	67.00 (27.0–98.0)	58.67 ± 12.7	66.00 (27.0–98.0)
Gender			
Male	152 (64.4%)	15 (50.0%)	167 (62.8%)
Female	84 (35.6%)	15 (50.0%)	99 (37.2%)
GLA Activity	4.02 (0.52–14.3) μmol/L wb/hr	17.13 ± 4.6 pmol/punch/hr	-
LVMI (g/m^2^)	129.01 (95.4–286.2)	130.25 (96.5–253.0)	129.01 (95.4–286.2)
LVIDd (cm)	4.42 ± 0.6	4.20 (3.5–6.4)	4.42 ± 0.6
LVIDs (cm)	2.90 (1.7–5.1)	2.86 ± 0.5	2.90 (1.7–5.1)
IVSd (cm)	1.40 (1.3–2.7)	1.79 ± 0.4	1.50 (1.0–2.7)

GLA indicates α-galactosidase enzyme; LVMI, left ventricular mass index; LVIDd, left ventricular internal dimension at end-diastole; LVIDs, left ventricular internal dimension at end-systole; IVSd, Interventricular septum thickness at end-diastole.

**Fig 2:**
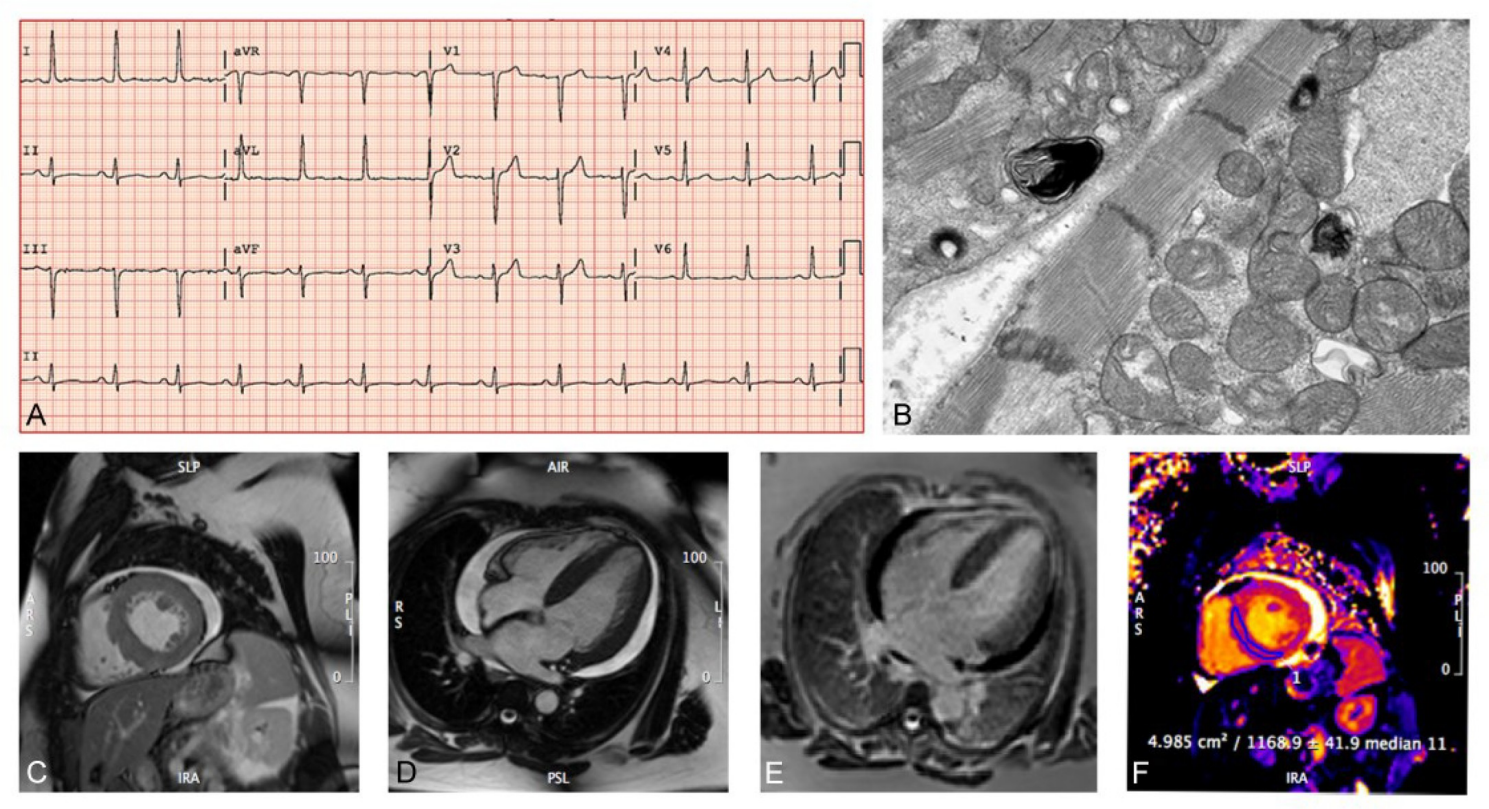
Phenotypic characterization of the patient with hypertrophic cardiomyopathy due to hydroxychloroquine-induced phenocopy. **(A)** Twelve-lead electrocardiogram showing elevated R wave in aVL and S wave in V3 consistent with LVH**. (B)** Endomyocardial biopsy and electron microscopy showing myeloid structures consistent with Fabry disease and other lysosomal storage diseases (magnification 10,000 x). **(C-F)** Cardiac magnetic resonance imaging showing bi-ventricular concentric hypertrophy, a moderate circumferential pericardial effusion, no convincing scar on LGE imaging and increased native myocardial T relaxation time (FD is characterized by decreased T1 time). LVH indicates Left Ventricular Hypertrophy.

The characteristics of the patients with FD are reported in Table 2. Our results show that 1.9% (5/266) of the patients with unexplained LVH who were screened with DBS testing were diagnosed with FD. The five patients with FD were all part of the Hong Kong cohort, males, had predictably lower α-galactosidase (α-GAL) enzyme activity, and all had the same mutation of the alpha-galactosidase gene (*GLA*) gene (IVS4 + 919G > A). There was no familial relationship between the 5 Hong Kong patients diagnosed with FD. We identified a 41-year-old female with hypertrophic cardiomyopathy (LVMI = 97 g/m^2^; LVIDd = 3.8 cm, and LVIDs = 2.4 cm) as a result of HCQ usage in the Edmonton cohort (Fig 2). The phenocopy was part of the Edmonton cohort, a female, who had normal α-GAL enzyme activity, and eight benign variants in her *GLA* gene. ECG findings showed distinct LVH based on voltage criteria with normal PR interval.

**Table 2:** Characteristics of patients with confirmed Fabry Disease (FD).

Case	Age	Gender	GLA activity (μmol/L wb/hr)	GLA gene	LVMI (g/m^2^)	LVIDd (cm)	LVIDs (cm)	FD
1	74	Male	0.55	IVS4 + 919G > A	222	5.2	3.3	Yes
2	59	Male	0.76	IVS4 + 919G > A	144	3.9	2.9	Yes
3	59	Male	0.52	IVS4 + 919G > A	147	4.1	2.6	Yes
4	62	Male	1.04	IVS4 + 919G > A	128	4.6	3.2	Yes
5	61	Male	1.15	IVS4 + 919G > A	208	4.9	2.9	Yes

GLA indicates α-galactosidase enzyme; LVMI, left ventricular mass index; LVIDd, left ventricular internal dimension at end-diastole; LVIDs, left ventricular internal dimension at end-systole; FD, Fabry Disease.

**Fig 3:**
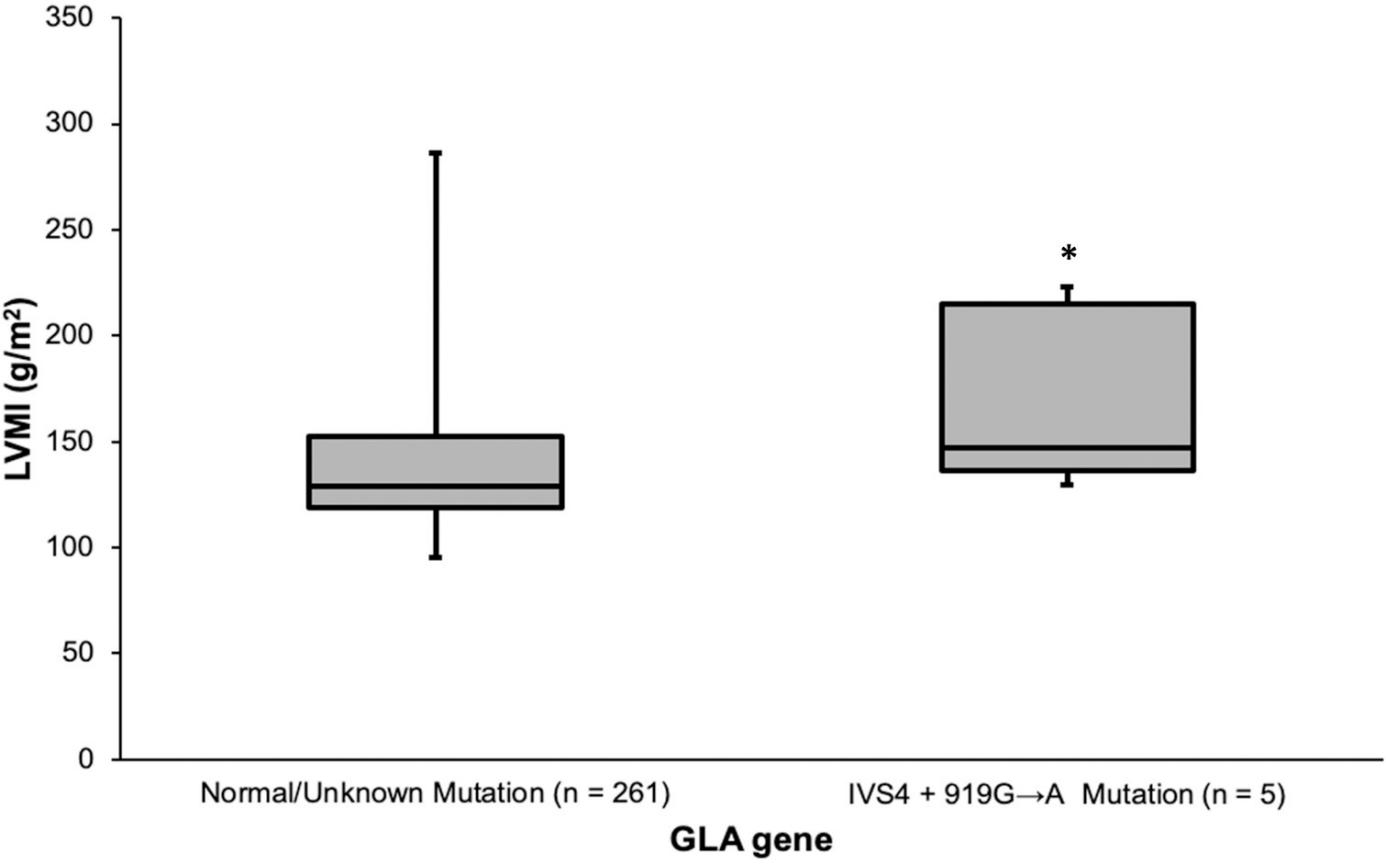
Comparing left ventricular mass index by *GLA* genotype. An independent-samples Mann-Whitney *U* Test was performed and revealed that LVMI is elevated in those patients with IVS4 + 919G > A mutations compared to those without a known mutation. LVMI indicates Left Ventricular Mass Index (g/m^2^); GLA, α-galactosidase gene. Whiskers represent maximum and minimum data points. *P<0.05.

**Fig 4:**
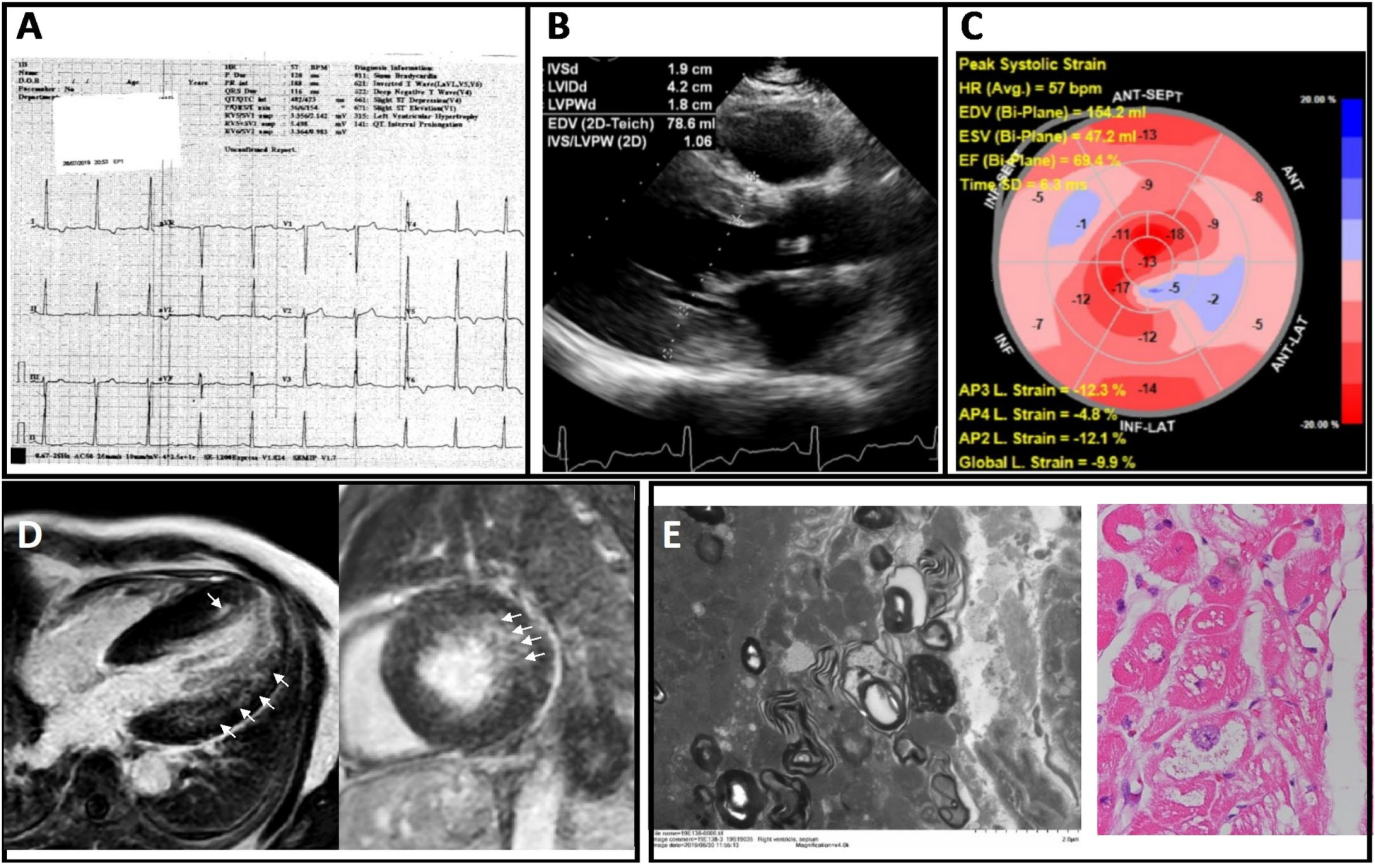
Electrocardiographic, imaging, and endomyocardial biopsy findings of a 60 year-old man originally diagnosed with nonobstructive hypertrophic cardiomyopathy. **(A)** Electrocardiography showed high voltage in limb and precordial leads consistent with LVH; **(B)** Transthoracic echocardiography showed LV walls thickening with maximum wall thickness of the septum and posterior wall measuring 1.7 cm and 1.9 cm respectively, with LV ejection fraction = 64%; **(C)** Global longitudinal strain was reduced (–9.9%); **(D)** Cardiac MRI showing long-axis (left) and short-axis (right) views illustrating concentric LVH and mild patchy late gadolinium enhancement in the lateral wall and the apical septum (arrows). **(E)** Pathological findings based on electron microscopy of endomyocardial biopsy showing central vacuolations and lamella bodies in cardiomyocytes (left) and PAS staining showing vacuolation and disarray (right), consistent with Fabry cardiomyopathy.

Linear regression analysis was performed to compare α-GAL enzyme activity against left ventricular mass index (LVMI), left ventricular internal diameter in diastole (LVIDd), and left ventricular diameter in systole (LVIDs) but no significant relationships were found. Comparison of the LVMI by *GLA* genotype showed that patients with the IVS4 + 919G > A mutation had higher LVMI compared with those without the mutation (Fig 3, *p* = 0.044). This relationship remained significant when the Hong Kong cohort was tested independently (*p* = 0.038). Three patients had cardiac MRI, which demonstrated asymmetric LVH in one patient; concentric basal hypertrophy with systolic anterior motion in another patient; and hypertrophy at basal and mid-ventricular inferoseptal wall and apical septum in a third patient with evidence of LVH and strain on 12-lead ECG (Fig 4). Of the 5 patients with genetically confirmed FD, 2 patients had endomyocardial biopsy, which showed myocytes with variable per-nuclear vacuolation with dot-like eosinophilic bodies (Fig 4). Of the 5 confirmed FD patients, two patients were considered eligible for ERT. One patient was considered not eligible for ERT based on his old age and advanced stage of disease. For the remaining two patients, clinical work up for ERT eligibility are currently in progress. For the 2 FD patients who received ERT, echocardiography showed regression in septal wall thickness, whereas in patients who were deemed ineligible or did not yet receive ERT, septal wall showed static or progressive septal wall thickening (Table 3).

**Table 3:** Demographic and clinical characteristics of patients with confirmed Fabry Disease (FD).

Age of diagnosis of FD (years)	Age of first presentation (years)	Initial presentation	ECG data	ERT	Baseline septal wall thickness (mm)	Septal wall thickness at 6 months after ERT commencement (mm)
74	71	Incidental murmur; aortic regurgitation	RBBB	No	19	19
59	54	Chest pain on exertion	LVH	Yes	19	18
59	59	Found LVH on echo during evaluation for AF	AF, nonspecific intraventricular conduction delay; inferior infarct	Yes	24	18.5
62	61	Atypical chest pain and recurrent syncope	AF, LVH; 2:1 AV block (requiring pacemaker)	No	13	17
61	42	Hypertension and LVH on echo	LVH	No	17	23

FD indicates Fabry Disease; ECG, electrocardiogram; ERT, enzyme replacement therapy; RBBB, right bundle branch block; LVH, left ventricular hypertrophy; AF, atrial fibrillation; AV, atrioventricular.

## Discussion

The traditional prevalence of FD was estimated at 1/100000, which may be an underestimation of its true prevalence since multiple large scale newborn and adult screening and cohort studies have shown a prevalence of approximately 1/3000 [4,24–26]. The clinical features of FD are heterogenous meaning patients present to various specialists with complex organ disorders leading to difficulties with diagnosis [27]. The delays in diagnosing FD can result in delayed treatment, despite enzyme replacement therapy being most beneficial when started early in the disease course [28]. DBS screening has been recommended as a large-scale screening tool for FD, since dried blood samples require less biological materials and enzyme activity is substantially more stable when compared to whole blood samples [29,30]. These characteristics make DBS an effective and easy to administer screening tool, which could be used to enhance and promote earlier diagnosis of FD amongst patients with unexplained or idiopathic left ventricular hypertrophy. There is also limited literature on the use of DBS testing in patients with unexplained LVH, so it was appropriate to investigate the clinical utility of DBS testing as a diagnostic tool in this patient population.

In our study, DBS testing and clinical assessment was able to successfully identify five patients with FD and one patient with hydroxychloroquine-induced phenocopy among our cohort of 266 patients with unexplained LVH. The prevalence of hypertrophic cardiomyopathy (HCM) in young adults in the United States has been estimated to be 1 in 500 [31]. Earlier screening studies of patients with late-onset hypertrophic cardiomyopathy found that 3% of Japanese males, 6.3% of UK males, and 12% of Italian females had FD [32–34]. However, recent studies suggest the prevalence to be lower. In a screening study of 2,034 probands, 37 (1.8%) were carriers of GLA mutations while cascade family screening identified 60 affected relatives with the heart being the organ most commonly involved [26]. A European multicenter study involving a large, consecutive cohort found that 0.5% of their patients with unexplained LVH had a mutation in their α-GAL enzyme [35]. Other work has also found a FD prevalence of approximately 1% in patients with unexplained HCM [36,37]. The results from our screening using DBS testing is comparable to these recent studies on the prevalence of FD in patients with undiagnosed LVH, as 1.9% of our patient cohort were diagnosed with FD. The results of this present study are novel, however, as there are limited data screening for FD in East Asian male and females with otherwise unexplained LVH. Two of these patients have now been initiated on enzyme replacement therapy. One patient was considered not eligible for ERT due to the advanced stage of his disease and old age, which clearly demonstrates the necessity for early identification of undiagnosed patients to allow for timely initiation of therapeutic interventions.

All patients with FD in our study were patients with a known intronic mutation, IVS4 + 919G > A. This mutation is documented to have a high frequency in Taiwan and mainland Chinese populations and has been linked to late-onset FD [38–41]. Up to 80% of FD incidence in Taiwan can be accounted for by this specific mutation. For this reason, it is logical that among our Hong Kong cohort, this was the only pathological variant that was noted in causing FD. Additionally, it was recently noted that all patients with the mutation outside of Japan were of Chinese origin, including in Australia, Canada, and the United States [41]. Since this mutation has a high prevalence amongst Asian populations, but not in Western populations; it can explain why we were unable to detect any patients with this mutation in the Edmonton cohort. However, the smaller size of our Edmonton cohort is an equally important factor to consider when interpreting these results. The IVS4 + 919G > A mutation causes alternative splicing in intron 4 causing the insertion of a 57-nucleotide sequence between exons 4 and 5 of the α-GAL gene leading to premature termination. A Chinese-Taiwanese neonatal screening study also revealed a high prevalence of mutations associated with cardiovascular variant FD, specifically the IVS4 + 919G > A mutation [38]. These alternatively spliced transcripts occur normally in most individuals, however, individuals with the IVS4 + 919G > A mutations will have a largely increased number of these alternatively spliced transcripts and a subsequently reduced enzyme activity as a result. Previous studies have identified this splicing mutation primarily amongst newborns and the identification of these 5 adult patients amongst our cohort should add to the growing body of literature on this specific mutation for FD. Recognizing this mutation in adults allows for the initiation of therapies and cascade family screening of immediate and extended family. The geographic and racial distribution of this mutation emphasizes a need for physicians to be aware of such disparities while constructing a differential diagnosis for patients with unexplained LVH.

HCQ was originally an anti-malarial drug that is now used commonly in the management of rheumatoid arthritis and lupus erythematosus and more recently in clinical trials for COVID-19 [42–45]. Cardiac toxicity is an under-recognized adverse effect of HCQ usage leading to cardiomyopathy with concentric hypertrophy, conduction abnormalities and prolonged QT interval resulting in increased morbidity and mortality with prolonged usage of the drug [46]. HCQ cardiomyopathy is a phenocopy of FD and an increased incidence of HCQ-induced myopathy have been reported in recent years [47–49]. We were able to identify the HCQ-induced phenocopy early on as a result of the screening of what appeared to be an idiopathic cause of cardiac hypertrophy. The patient was withdrawn from HCQ immediately upon recognition and after 18 months her LVMI decreased from 97 g/m^2^ to 82 g/m^2^. Her left atrial size and diastolic function have normalized as well. These results emphasize the need for early recognition of HCQ-induced cardiomyopathy and the need for both general practitioners and specialists to be aware of the adverse effects of the drug on cardiovascular health.

## Limitations and future directions

This current study is limited by the fact that sample sizes across Edmonton and Hong Kong cohorts were not equal. However, both populations were similar in terms of left ventricular parameters and other demographic characteristics. All patients with confirmed FD had the same the IVS4+919G > A mutation, which has been noted as being a founder mutation that originated in mainland China more than 800 years ago [41]. Therefore, failure to identify a similar mutation in the Edmonton cohort is related to the smaller size of the Edmonton cohort and the geographic distribution of this mutation. Finally, although this study does have novel contributions, we acknowledge that this one of several studies describing the prevalence of FD in a cohort of patients with otherwise unexplained LVH.

In future studies, it would be useful to compare gene variants of unknown significance (GVUS) to left ventricular parameters to identify whether certain benign variants may in fact be pathogenic and associated with lower α-GAL enzyme activity. Another future direction includes performing a prospective screening study where DBS testing is utilized as a gatekeeper to other more invasive and/or costly procedures, such as cardiac MRI and endomyocardial biopsy, in patients with unexplained or idiopathic hypertrophic cardiomyopathies. Additionally, a health-economic analysis on the use of DBS testing in the diagnosis of FD and other genetic conditions may be warranted to determine whether DBS testing is more cost-effective than other genetic testing modalities.

## Conclusions

This study highlights the importance of early screening for FD in patients with unexplained LVH, due to the availability of effective treatment options for those receiving an early diagnosis of FD. Additionally, the initiation of targeted therapies for these patients outlines a potential role for DBS testing in advancing the utilization of precision medicine in the diagnosis and treatment of cardiovascular disease. Therefore, this study supports the use of DBS testing as an effective and easy to administer screening tool to carry out on patients with undiagnosed LVH.

## Supporting information

S1 File Minimal anonymized raw data for dried blood spot screening study in Hong Kong and Edmonton. (XLSX)
